# Room-Temperature CMOS Monolithic Resonant Triple-Band Terahertz Thermal Detector

**DOI:** 10.3390/mi14030627

**Published:** 2023-03-09

**Authors:** Xu Wang, Ting-Peng Li, Shu-Xia Yan, Jian Wang

**Affiliations:** 1 State Key Laboratory of Complex Electromagnetic Environmental Effects on Electronics and Information System, Luoyang 471003, China; 2 School of Electronics and Information Engineering, Tiangong University, Tianjin 300387, China; 3 School of Microelectronics, Tianjin University, Tianjin 300072, China; 4 Qingdao Institute for Ocean Technology, Tianjin University, Qingdao 266200, China

**Keywords:** terahertz, multiband detector, CMOS, octagonal ring antenna

## Abstract

Multiband terahertz (THz) detectors show great application potential in imaging, spectroscopy, and sensing fields. Thermal detectors have become a promising choice because they could sense THz radiations on the whole spectrum. This paper demonstrates the operation principle, module designs with in-depth theoretical analysis, and experimental validation of a room-temperature CMOS monolithic resonant triple-band THz thermal detector. The detector, which consists of a compact triple-band octagonal ring antenna and a sensitive proportional to absolute temperature (PTAT) sensor, has virtues of room-temperature operation, low cost, easy integration, and mass production. Good experimental results are obtained at 0.91 THz, 2.58 THz, and 4.2 THz with maximum responsivities of 32.6 V/W, 43.2 V/W, and 40 V/W, respectively, as well as NEPs of 1.28 μW/Hz^0.5^, 2.19 μW/Hz^0.5^, and 2.37 μW/Hz^0.5^, respectively, providing great potential for multiband THz sensing and imaging systems.

## 1. Introduction

Terahertz (THz) waves possess many unique properties, such as penetrating nonconductive materials, which are opaque in visible and infrared bands [1]. They could identify specific materials according to their characteristic THz signatures [2]. THz waves are safe for biological tissue because of low photon energy and non-ionizing attributes, in contrast to X-rays, and promise higher resolution compared with microwave bands [3,4]. The above characteristics of THz waves have promoted THz technology to make great progress in medical detection [5], security inspection [6], non-destructive testing [7], wireless communication [8], atmospheric monitoring [9], astronomical observation [10], and so on. However, the interest in a wide range of commercial THz applications is the main driver for the development of widely accessible room-temperature THz detectors. In addition, as the same material is imaged at different frequencies, the image sharpness would vary with different transmission rates. Hence, multiband detectors could dramatically improve the overall sensing and imaging ability by means of obtaining more informative images through fusion technology [11]. Besides, multiband detectors also have the advantages of enhanced detection probability, increased calibration capability, and reduced influences of standing waves or scattering, showing great potential for further development of THz applications [11,12,13,14].

With continuous developments in CMOS technology, which is considered as an attractive device technology because of its low cost, high yield, and high integration ability [15], various kinds of room-temperature CMOS multiband THz detectors have attracted more attention and have gradually received in-depth research in the last couple of years [16,17,18,19,20]. Multiband active detectors use a higher harmonic, resulting in sharply increased noise figures and fixed operation frequencies, which are determined by fundamental and harmonic frequencies [21]. Similar trends do not exist for passive devices that are more suitable for human vision and image processing [22]. Therefore, several multiband passive detectors consisting of antennas and MOSFETs have been proposed [16,17,18,19,20]. The above FET-based THz detectors, whose operation frequencies are seriously restricted and influenced by transistors, achieve better characteristic results below 1 THz, and their performances degrade dramatically as the frequency exceeds 1 THz owing to frequency-dependent parasitic elements [19]. Besides, the detectors in [19,20] are composed of multiple discrete antennas and multiple FETs, resulting in lower integration levels, a larger chip area, and higher cost, thus it is necessary to design compact multiband THz detectors. However, it is hard to obtain compact FET-based detectors because the input impedance of transistors differs at multiple frequencies [16,21]. Compared with FET-based detectors, THz thermal detectors constitute promising options as they allow wideband detection, support high-frequency THz detection, and show performance advantages in higher THz bands because their output signals are independent of frequencies [23,24,25]. Therefore, several room-temperature CMOS multiband THz thermal detectors have been proposed. A room-temperature CMOS multiband THz thermal detector composed of an antenna and an NMOS sensor is proposed, but it operates at 0.546 THz, 0.688 THz, 0.78 THz, and 0.912 THz [26]. It is necessary to design room-temperature CMOS multiband THz thermal detectors that could detect sub 1 THz waves and above 1 THz waves to possess good sensitivity and high resolution [11]. Previous works have described two kinds of CMOS triple-band THz thermal detectors, which mainly concentrate on modules’ designs, including designs of receiving structures and temperature sensors, thus they lacked the concept of collaborative designs between modules, such as completing the layout of a temperature sensor according to the raised temperature distribution of receiving structures [27,28]. Besides, these triple-band detectors just completed the performance characterizations at two frequencies.

This paper presents a compact room-temperature triple-band THz thermal detector made up of a strong octagonal ring antenna and a sensitive PTAT sensor using a Global Foundry 55 nm CMOS process. Because lower THz waves have a greater penetration depth and higher THz waves provide better spatial resolution, the proposed detector is chosen to operate at 0.91 THz, 2.58 THz, and 4.2 THz for available THz sources so as to obtain better penetration and greater spatial resolution. It achieves relatively better measurement results at three operation frequencies with detailed analysis, exactly presenting an uncooled, compact, cost-effective, easy-integration, and mass-production multiband detection system.

## 2. Detector Structure and Operation Principle

**Figure 1:**
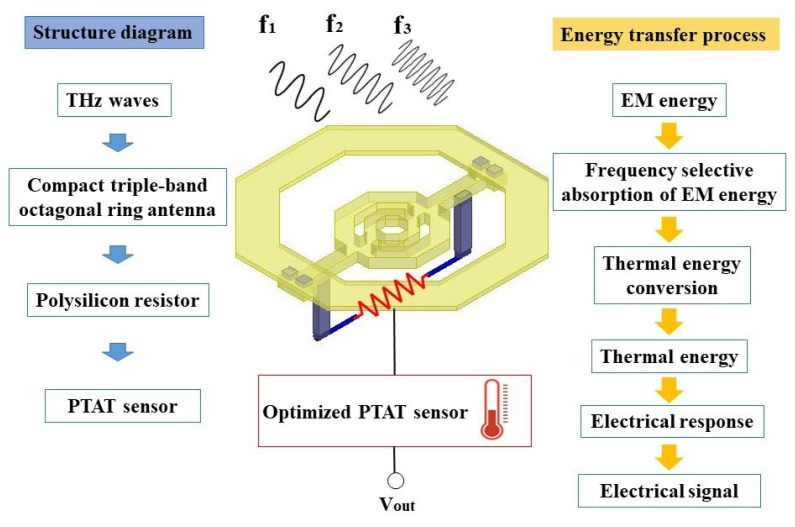
Schematic diagram of the proposed triple-band detector.

Antennas are ready to shape the radiation pattern and tune the impedance match within a wider bandwidth [29], while octagonal rings are used to constitute antennas because they have advantages of smaller chip area occupation and less coupling effect than other structures [30]. In addition, PTAT sensors as a type of common CMOS temperature sensor show great application potential owing to their better linearity and accuracy [31]. Based on this, Figure 1 shows the schematic diagram of the proposed detector, which consists of a compact triple-band octagonal ring antenna, a polysilicon resistor at the termination of the antenna, and a sensitive PTAT sensor. As THz waves interact with the antenna, an instantaneous frequency-dependent current is excited and flows through the resistor; by this means, incident THz waves are frequency-selective absorbed. Thus, electromagnetic (EM) energy is immediately transformed into thermal energy through ohmic loss and conductive loss, leading to the localized temperature increment depending on the magnitude of the radiation [24]. In addition, the PTAT sensor transforms the rising temperature into an increased output voltage, so the sensor is located below the antenna and in close proximity to the resistor in order to reduce heat loss and sense an increased temperature as fast as possible. The triple-band detection is accomplished as THz waves of three frequencies are incident on the detector successively.

Furthermore, a lower operating frequency leads to larger antenna sizes, so the temperature distribution caused by conductor loss is far from that caused by ohmic loss. The resistor becomes the main heat source and presents a strong, uniform, and raised temperature distribution in a certain area, because temperature sensing elements of the sensor should sense the same temperature and the received EM energy is mainly converted into joule heat through the resistor. Therefore, the increased temperature is approximately equal to the temperature increment generated by the resistor. However, a higher operating frequency leads to smaller antenna sizes, so the temperature distribution generated by the antenna and the resistor is closed or even overlapped and, finally, a strong, uniform, and raised temperature distribution is generated in a certain area centered on the resistor. In this way, the perceived temperature increment is approximately equal to the sum temperature increment caused by the resistor and the conductor of the antenna.

According to the operation principle of the detector, its design task not only includes the independent design of the antenna and the PTAT sensor, but also contains the co-design between the antenna and the PTAT sensor based on the temperature distribution of the antenna caused by the incident THz waves.

### 2.1. Design of Octagonal Ring Antenna

**Figure 2:**
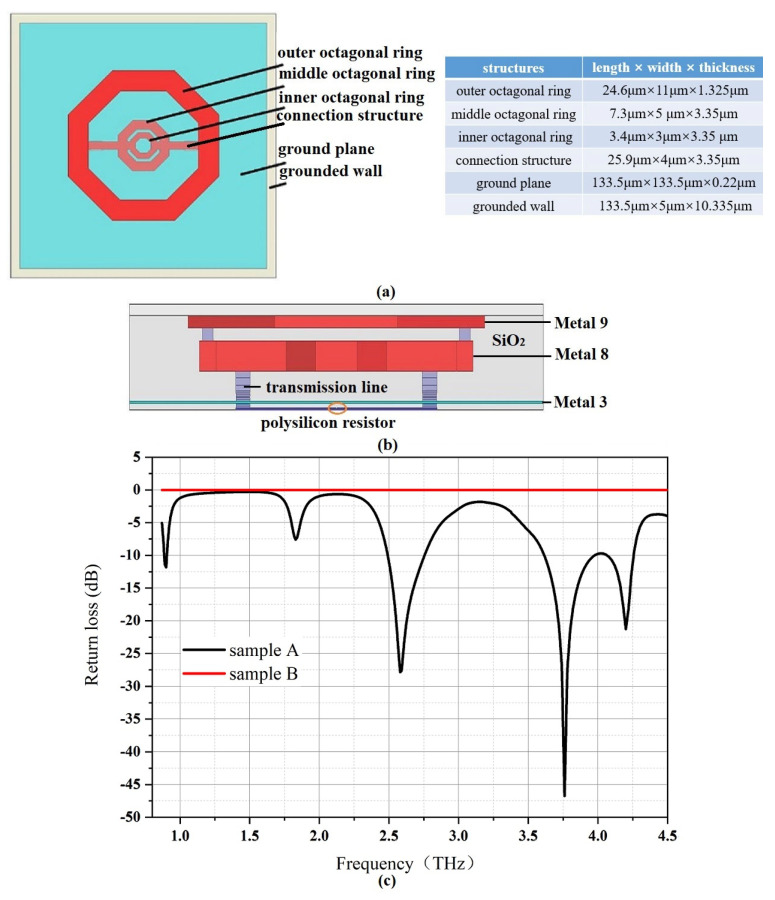
Designed antenna: (**a**) top view; (**b**) side view; (**c**) simulated return loss.

The structure diagram and optimized geometric parameters of a compact triple-band octagonal ring antenna (sample A) using HFSS tools are shown in Figure 2a,b. Nested octagonal rings are composed of three concentric rings with different sizes, and the smaller ring is embedded in the larger ring. As perimeters of the outer ring, the middle ring, and the inner ring are about dielectric wavelengths of 0.91 THz waves, 2.58 THz waves, and 4.2 THz waves, respectively, the fundamental modes of the outer octagonal ring, the middle octagonal ring, and the inner octagonal ring could radiate 0.91 THz waves, 2.58 THz waves, and 4.2 THz waves, correspondingly. Based on the reciprocity theorem, sample A could also receive THz waves of 0.91 THz, 2.58 THz, and 4.2 THz, respectively.

As shown in Figure 2a,b, sample A was made up of three nested octagonal rings, connection structures, a ground plane, two transmission lines, and a grounded wall. The outer octagonal ring was constructed in the metal 9 layer of the 55 nm CMOS process, while the middle and inner octagonal rings were fabricated in the metal 8 layer. The connection structures between the outer octagonal ring and the middle octagonal ring were realized in metal 8. The metal 3 layer was used to fabricate the metallic ground plane, which could effectively prevent the waves from being exposed to the lossy substrate because metal 1 and metal 2 were used for the electronics routing of the PTAT sensor. The resistance of the polysilicon resistor was 100 Ω for impedance matching and transmission lines were formed from the octagonal ring antenna down to the resistor. A grounded wall composed of metal layers and vias layers around the antenna was applied to trap EM energy and prevent external interference. Besides, metal layers and inter-metal dielectric regions were modeled as aluminum and SiO_2_, respectively, and they were fixed by the CMOS process. In addition, sample B with only a ground plane in the metal 3 layer was also simulated for verifying the frequency-selective absorption of sample A.

Figure 2c shows the simulated return loss curves, where sample A could resonate at 0.91 THz, 2.58 THz, and 4.2 THz, while sample B does not have frequency−selective characteristics. Within the observation frequency range, three octagonal rings radiate 0.91 THz waves, 2.58 THz waves, and 4.2 THz waves, while the second-order mode and fourth-order mode of the outer octagonal ring correspond to radiating 1.82 THz waves and 3.74 THz waves, respectively. Although sample A could radiate THz waves of five frequencies, it is still considered that a triple-band THz antenna is obtained instead of a five-band antenna. Besides, the fundamental modes of three octagonal rings are still applied to radiate 0.91 THz waves, 2.58 THz waves, and 4.2 THz waves, instead of only constructing an outer octagonal ring to obtain a THz antenna operating in multiple bands through its fundamental modes and higher modes. This is because, compared with fundamental modes, higher order modes of the antenna are unstable, and have less energy and greater loss. In addition, as the antenna operates with higher order modes, the directional pattern usually has a large domain change because of the multi-periodic current distribution. Therefore, the application of higher order modes of the antenna is generally not recommended. As sample A obtains simulated gains of 3.9 dBi, 4.24 dBi, and 3.13 dBi in the *z*-axis direction with simulated radiation efficiencies of 63.5%, 82.6%, and 83.4% at 0.91 THz, 2.58 THz, and 4.2 THz, respectively, the receiving efficiencies of sample A towards 0.91 THz waves, 2.58 THz waves, and 4.2 THz waves are 63.5%, 82.6%, and 83.4%, respectively.

### 2.2. Design of the PTAT Sensor

**Figure 3:**
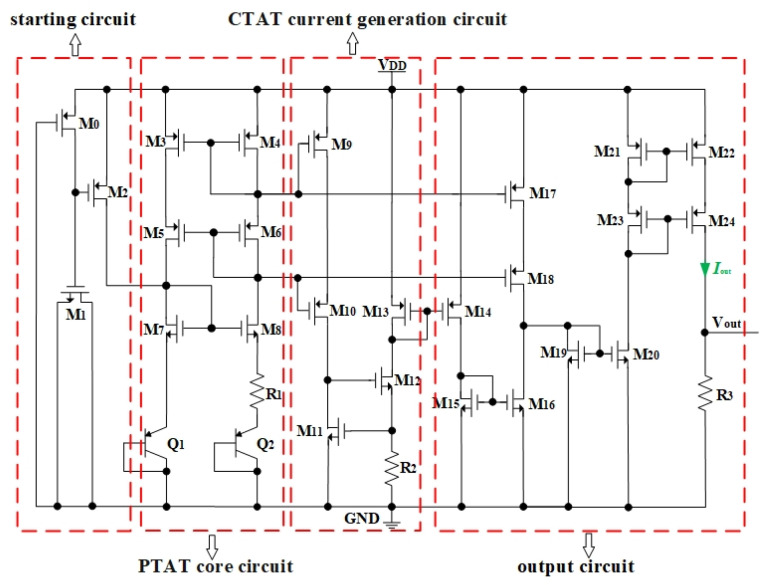
Schematic diagram of the optimized PTAT sensor.

Bipolar junction transistors (BJTs) have become attractive temperature sensing elements because of their lower cost, better stability, higher temperature sensitivity, lower power consumption, and better process compatibility and predictability [32]. Besides, it was recognized that, if two BJTs with the same emitter area operated at different current densities or two BJTs with proportional emitter areas operated at the same current density, then their emitter–base difference voltage is proportional to the absolute temperature [33]. Therefore, PTAT sensors that convert the temperature increment into an output voltage variation proportionally constitute a type of circuit structure that relies on BJTs’ temperature characteristics [34]. In order to obtain detectors with higher responsivity, there is an effective approach to adopt an optimized PTAT sensor made up of four modules with enhanced temperature sensitivity. Previous research has proposed a sensitive PTAT sensor with an increased temperature sensitivity of 10.31 mV/°C at 25 °C, as shown in Figure 3 [27]. The starting circuit is used to ensure the sensor quickly enters the normal operation state at the moment of supplying power. The PTAT current *I*_PTAT_ could be generated by the PTAT core circuit, and the complementary to absolute temperature (CTAT) current *I*_CTAT_ could be obtained from the CTAT current generation circuit. *I*_PTAT_ and *I*_CTAT_ are differentiated in a proper proportion to obtain a PTAT current with an enhanced positive temperature coefficient in the output circuit and, finally, a PTAT voltage is generated.

### 2.3. Co-Design of Antenna and PTAT Sensor

As 0.91 THz waves are incident normally, under the action of electric field horizontally to the right along xoy plane, the right side of the outer octagonal ring would gather a large amount of positively induced charge, while the left side would gather equal amounts of negatively induced charge. By this means, the outer octagonal ring generates an induced electromotive force (EMF) and an induced electric field, which is opposite to the incident electric field of 0.91 THz waves. As the circumference of the outer octagonal ring is about a dielectric wavelength of 0.91 THz waves, accumulated anisotropic charges cause an induced current distributed in full wave among the outer octagonal ring, and the current finally flows to the resistor through feeding structures. Similarly, as 2.58 THz waves or 4.2 THz waves are incident normally, the middle octagonal ring or inner octagonal ring would generate an induced electric field and an induced current, which also flows to the resistor. It could be concluded that octagonal rings and the resistor constitute heat sources. Therefore, the temperature distribution of the antenna is obtained using the EM field frequency domain module and solid heat transfer module in COMSOL tools. The lumped port and boundary conditions are set to make sure that the antenna could frequency-selective receive THz waves, and incident power at the excitation port is set according to the receiving efficiency enabling the antenna to receive EM energy of 0.1 mW. Besides, the antenna and resistor are set as heat sources, and the initial temperature of the model is set to 25 °C. The temperature distribution of the antenna is obtained by frequency domain research and steady state research.

**Figure 4:**
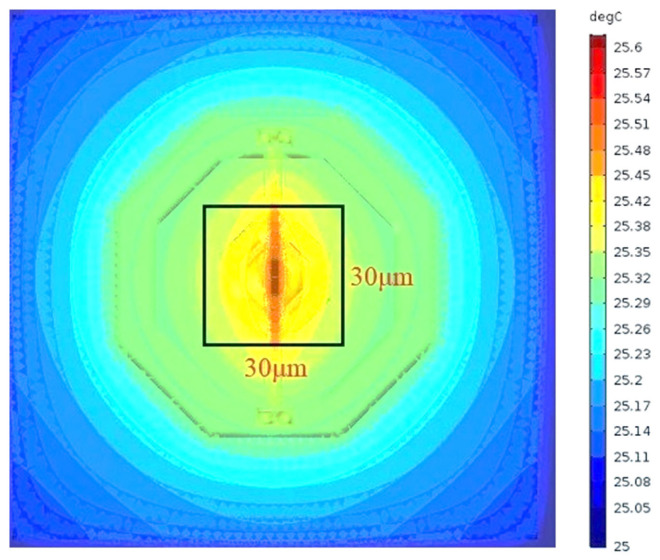
Temperature distribution of the proposed detector.

In order to clarify the layout of temperature-sensing elements and determine whether all temperature-sensing elements could be distributed within the same raised temperature distribution area, the raised temperature distribution of the antenna needs to be simulated. As the antenna receives 0.91 THz waves, the resistor at the termination of the antenna becomes the main heat source owing to ohmic loss, showing a strong temperature distribution in a certain area around the resistor. According to the antenna size, the certain area could be approximately considered as 30 μm × 30 μm, and this means that the resistor generated a strong, uniform, and raised temperature distribution in this area, as shown in Figure 4.

Then, 2.58 THz waves or 4.2 THz waves with the same EM energy of 0.1 mW were incident on the antenna, so the resistor that acts as the main heat source also generated a strong and uniform temperature in this area. Based on this, two BJTs of the PTAT sensor are the main temperature-sensing elements, and the transistor M_11_ working in the subthreshold region could also sense the raised temperature. Thus, two BJTs and M_11_ should be distributed within this area, which is centered around the resistor with an area of 30 μm × 30 μm. In this way, it not only benefits sensing the raised temperature effectively, but also could ensure that all temperature-sensing elements sense the same temperature. In addition, two BJTs are composed of a single transistor unit and seven parallel transistor units with an emitter area of 5 µm × 5 µm and an overall size of 10 µm × 10 µm. Besides, the overall size of M_11_ is 3 µm × 25 µm. It could be seen that, as two BJTs are closely arranged in a “square” shape, and there is an empty position of one transistor unit in the center to construct a polysilicon resistor, BJTs could be centrally arranged around the resistor. At the same time, when transistor M_11_ is distributed near two BJTs, the layout area formed by temperature-sensing elements is basically consistent with the temperature distribution generated by the antenna.

## 3. Experiment and Discussions

**Figure 5:**
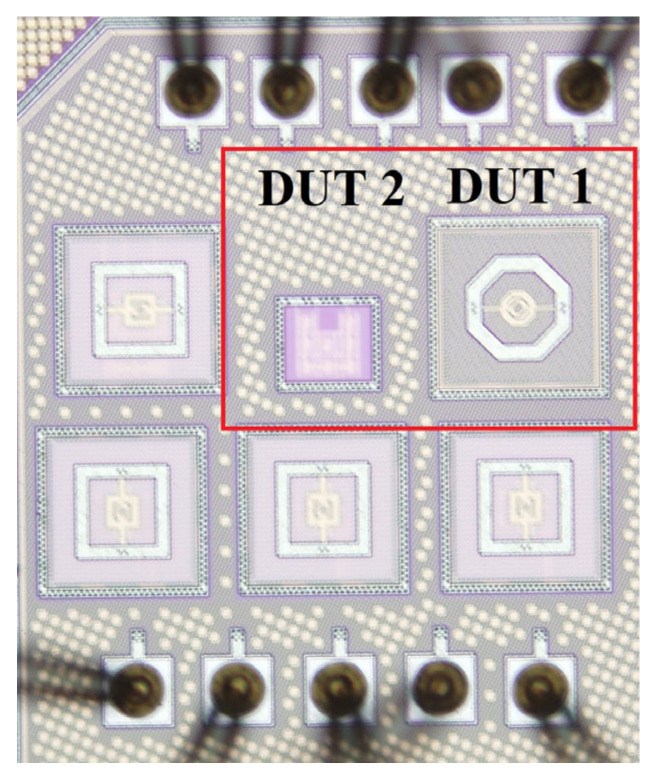
Die micrograph of thermal detectors.

A die micrograph of the designed detector (DUT 1) and a device with only a PTAT sensor (DUT 2) is shown in Figure 5. DUT 1 and DUT 2 occupy chip areas of 164 μm × 214 μm and 141 μm × 193 μm, respectively. Various figures of merit, such as responsivity, noise equivalent power (NEP), and thermal time constant, are adopted to evaluate the performances of detectors.

### 3.1. Responsivity

The responsivity (*R*_v_) was determined by the output voltage difference Δ*V_out_* and the incident power *P_in_*. Δ*V_out_* was obtained by monitoring output voltages of detectors while THz waves were on and off. *P_in_* was calculated by the effective area *A_eff_* of the antenna and incident power density *J_in_*, which was obtained by the total power of the THz beam and the area of the focused beam. *R_v_* and *A_eff_* could be expressed as follows [35]:

(1) $$R_{v}=\frac{\Delta V_{out}}{P_{in}}=\frac{\Delta V_{out}}{J_{in}\cdot A_{eff}}$$

(2) $$A_{eff}=\frac{G\cdot \lambda^{2}}{4\pi }$$

where *G* is the gain and *λ* is the EM wavelength in free space.

**Figure 6:**
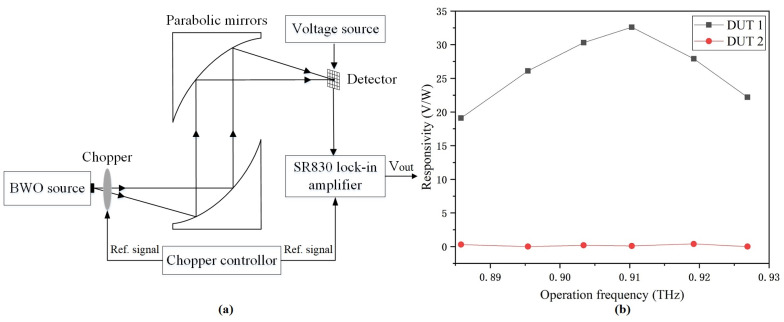
(**a**) Output voltage measurement setup as detectors operate at 0.91 THz. (**b**) Responsivities.

The block diagram of the output voltage measurement setup at 0.91 THz is shown in Figure 6a. The backward-wave oscillator (BWO) radiated THz waves with an average power of about 125 μW at 0.91 THz. Adjusting the height and position of two parabolic optical mirrors, as the THz beam was reflected by the first parabolic mirror, almost all THz waves were collimated and incident to the parallel second parabolic mirror. Then, after reflection and focusing, a THz beam with a diameter of about 1.15 mm was obtained. Detectors mounted on a three-dimensional stage were positioned at the focus point of the THz beam. A mechanical chopper with a minimum chopping frequency of 2 Hz modulated the THz beam, and a chopper controller modulated the chopper and the lock-in amplifier synchronously. The detector was biased at 2.5 V using a DC voltage source, and the output voltage was measured by a lock-in amplifier. As shown in Figure 6b, DUT 1 achieved the highest responsivity of 32.6 V/W as it resonated at 0.91 THz with a chopping frequency of 2 Hz. The responsivity at 0.91 THz was higher than the responsivity at both sides of 0.91 THz for the following reasons. On the one hand, the EM modeling of the antenna fully considered the design rules and implementation methods of the process in order to ensure that the antenna model was highly consistent with the layout. On the other hand, the circumference of the outer octagonal ring constructed by the metal 9 layer determined the operation frequency, and the processing tolerance of the metal 9 layer was ±0.135 μm. However, even with the maximum processing tolerance, the receiving of 0.91 THz waves was still higher than the receiving of THz waves on both sides of 0.91 THz. Furthermore, the responsivity of DUT 2 was close to zero as there was no significant voltage variation while THz waves were on or off.

**Figure 7:**
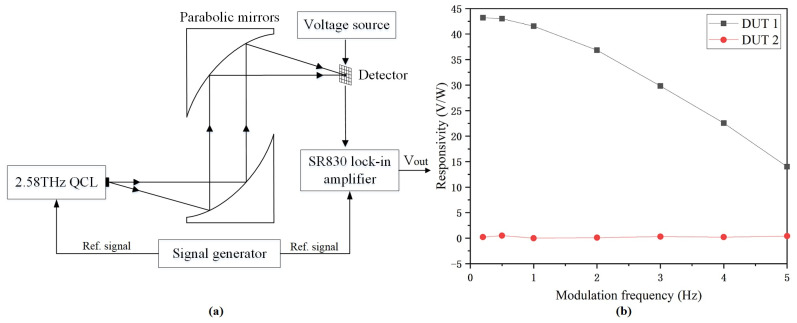
(**a**) Output voltage measurement setup as detectors operate at 2.58 THz. (**b**) Responsivities.

Figure 7a shows the output voltage measurement setup at 2.58 THz. A quantum cascade laser (QCL) radiated 2.58 THz waves with a peak power of 60 mW and a duty cycle of 4%. The 2.58 THz beam with a diameter of about 500 μm was collimated and focused using two parabolic optical mirrors. The detector was biased at 2.5 V and the output voltage was measured by the SR830 lock-in amplifier. The responsivity as a function of modulation frequency was acquired by modulating the QCL and the amplifier simultaneously using a signal generator. Figure 7b shows the measured responsivities of DUT 1 and DUT 2 versus modulation frequencies. Because the duration of THz waves on detectors increased as the modulation frequency decreased, the output voltage and responsivity of detectors gradually increased until the output was saturated. DUT 1 almost reached saturation at the modulation frequency of 0.5 Hz with a responsivity of 43.2 V/W, while DUT 2 showed responsivities of near zero.

**Figure 8:**
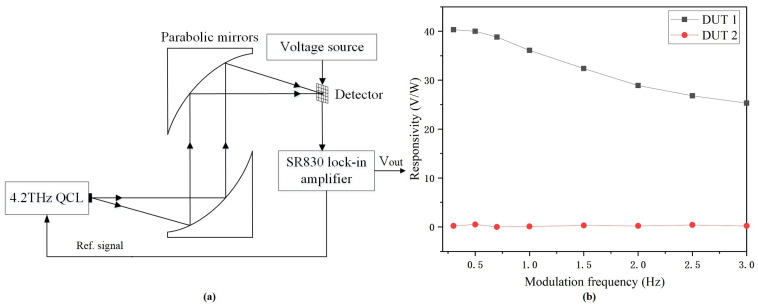
(**a**) Output voltage measurement setup as detectors operate at 4.2 THz. (**b**) Responsivities.

Figure 8a shows the output voltage measurement setup at 4.2 THz. A QCL provided 4.2 THz waves with an average power of about 0.5 mW and a duty cycle of 40%. The THz beam with an average power of about 0.15 mW and a diameter of about 209 μm was incident on the chip because of the strong absorption of 4.2 THz waves. The SR830 lock-in amplifier outputs synchronous modulation signals to the QCL and the output signal of the detector simultaneously. The responsivity as a function of modulation frequency from 0.3 Hz to 3 Hz was obtained from the lock-in amplifier as the detector was biased at 2.5 V. Figure 8b shows the measured responsivities of DUT 1 and DUT 2. DUT 1 almost reached saturation at the modulation frequency of 0.5 Hz with a responsivity of 40 V/W, while DUT 2 showed responsivities of near zero.

### 3.2. NEP

NEP was equal to the noise spectral density (NSD) divided by the responsivity. In addition, the NSD was obtained by measuring noise voltages using a dynamic signal analyzer as detectors were biased at 2.5 V [36]. As the proposed detector operated at 0.91 THz with a modulation frequency of 2 Hz, it obtained an NEP of 1.28 μW/Hz^0.5^. Besides, as the detector operated at 2.58 THz and 4.2 THz, it almost reached saturation at a modulation frequency of 0.5 Hz, thus corresponding NEPs were 2.19 μW/Hz^0.5^ and 2.37 μW/Hz^0.5^, respectively.

### 3.3. Thermal Time Constant

The thermal time constant represented the duration for the temperature change and output signals rising from 0 to 63.2% of the steady-state values, deriving from the responsivity versus modulation frequency [36]. The thermal time constant values of the proposed detector operating at 2.58 THz and 4.2 THz were extracted as 298 ms and 330 ms, respectively.

### 3.4. Performance Summary and Comparison

Although multiband thermal detectors show lower responsivity and higher NEP than multiband semiconductor detectors, they exactly constitute compact multiband structures, allow a wide spectrum detection, and avoid multiband impedance matching. Therefore, the proposed triple-band THz thermal detector and several published thermal detectors are summarized and compared in Table 1. A quad-band thermal detector consisting of an antenna and an NMOS sensor is designed to operate at 0.546 THz, 0.688 THz, 0.78 THz, and 0.912 THz [26]. It obtains better characteristic results, but operation at higher frequencies is preferable, thus THz thermal detectors operating above 1 THz have been proposed. Thermal detectors operating at three single frequencies of 1 THz, 2.9 THz, and 28.3 THz are composed of three discrete detectors with worse characteristic results [24]. In addition, compared with triple-band detectors in [27,28], the proposed detector applied an octagonal ring antenna as an alternative absorbing structure, showing a slight difference in performance due to the processing error or measurement error. Besides, the proposed detector, which was measured at three operation frequencies, also highlighted the concept of collaborative designs and detailed analysis. Although performance measurements of the proposed detector were finished at three operation frequencies, we would like to obtain traces of the output voltage from an oscilloscope or source meter at a certain chopping frequency for the purpose of visualizing in-time performances greatly in the future. Furthermore, in future work, we also prefer to compare performances at different ambient temperatures in order to further estimate the best possible performances.

## 4. Conclusions

To conclude, this paper presents the design, simulation, and experimental validation of a room-temperature monolithic resonant triple-band THz thermal detector, which was fully implemented in a CMOS process, allowing reduced fabrication complexity and lower production cost. The detector was composed of a compact triple-band octagonal ring antenna loaded with a polysilicon resistor and a sensitive PTAT sensor. The responsivity, noise equivalent power, and thermal time constant of the detector were experimentally assessed at 0.91 THz, 2.58 THz, and 4.2 THz, showing relatively better measurement results. The detector also has natural scalability to focal plane arrays, demonstrating significant advances in developing compact, room-temperature, low-cost, and mass-production multiband THz detection systems.

## Figures and Tables

**Table 1:** Performance summary and comparison.

Ref. No	Frequency (THz)	Structure	Technology	Rv (V/W)	NEP (W/√Hz)
[26]	0.546, 0.688, 0.78, 0.912	Antenna + NMOS sensor	0.18 μm CMOS	5.5 k, 5.3 k, 3 k, 5 k	0.94 p, 0.98 p, 1.72 p, 1.03 p
[24]	1, 2.9, 28.3	Dipole antenna + PTAT sensor	0.18 μm CMOS	18 *, 18.9, 18.6 *	1.7 μ *
[27]	0.91, 2.58, 4.3	Metamaterial absorber + PTAT sensor	55 nm CMOS	33.4, 47.9, 61.11 *	1.49, 1.88, 1.31 * μ
[28]	0.91, 2.58, 4.3	Loop antenna + PTAT sensor	55 nm CMOS	29.2, 46.5, 47.6 *	1.57, 1.26, 3.29 * μ
This work	0.91, 2.58, 4.2	Octagonal ring antenna + PTAT sensor	55 nm CMOS	32.6, 43.2, 40	1.28, 2.19, 2.37 μ

* based on simulation results.

## Data Availability

Not applicable.
